# Dopaminergic signaling supports auditory social learning

**DOI:** 10.1038/s41598-021-92524-1

**Published:** 2021-06-23

**Authors:** Nihaad Paraouty, Catherine R. Rizzuto, Dan H. Sanes

**Affiliations:** 1 Center for Neural Science, New York University, 4 Washington Place, New York, NY 10003 USA; 2 Department of Psychology, New York University, New York, NY 10003 USA; 3 Department of Biology, New York University, New York, NY 10003 USA; 4 Neuroscience Institute, NYU Langone Medical Center, New York University, New York, NY 10003 USA

**Keywords:** Social behaviour, Social neuroscience, Learning and memory

## Abstract

Explicit rewards are commonly used to reinforce a behavior, a form of learning that engages the dopaminergic neuromodulatory system. In contrast, skill acquisition can display dramatic improvements from a social learning experience, even though the observer receives no explicit reward. Here, we test whether a dopaminergic signal contributes to social learning in naïve gerbils that are exposed to, and learn from, a skilled demonstrator performing an auditory discrimination task. Following five exposure sessions, naïve observer gerbils were allowed to practice the auditory task and their performance was assessed across days. We first tested the effect of an explicit food reward in the observer’s compartment that was yoked to the demonstrator’s performance during exposure sessions. Naïve observer gerbils with the yoked reward learned the discrimination task significantly faster, as compared to unrewarded observers. The effect of this explicit reward was abolished by administration of a D1/D5 dopamine receptor antagonist during the exposure sessions. Similarly, the D1/D5 antagonist reduced the rate of learning in unrewarded observers. To test whether a dopaminergic signal was sufficient to enhance social learning, we administered a D1/D5 receptor agonist during the exposure sessions in which no reward was present and found that the rate of learning occurred significantly faster. Finally, a quantitative analysis of vocalizations during the exposure sessions suggests one behavioral strategy that contributes to social learning. Together, these results are consistent with a dopamine-dependent reward signal during social learning.

## Introduction

A broad range of behavioral paradigms demonstrate that *explicit* rewards, such as food or money, can reinforce behaviors and facilitate learning^1–10^. These reinforcing rewards typically engage dopaminergic signaling which acts to modulate both motivation and memory formation^11–17^. Specifically, dopamine neuron activity in pars compacta of substantia nigra and the ventral tegmental area is correlated with reward presentation, as well as reward anticipation^18, 19^. Furthermore, anticipatory dopaminergic activity is thought to guide and accelerate learning through D1 dopamine receptor-dependent long-term potentiation^14, 20, 21^. Reward anticipation can also enhance attention and, in turn, boost the encoding of incoming sensory signals^22–24^. Thus, explicit rewards have a positive impact on the learning and retention of sensory and motor skills^11, 25–29^.

The role of dopaminergic signaling in explicit reward learning suggests that dopamine may also be engaged by *implicit* rewards, such as those triggered by learning in the absence of any external feedback^30, 31^ or certain intrinsic motivational states such as curiosity^32^. In principle, implicit reward signals share some of the neural mechanisms that attend the acquisition of an external reward, including dopamine release in the nucleus accumbens^33–36^. For example, fluctuations in dopamine levels occur in rat nucleus accumbens when naïve animals observe the delivery of an explicit reward to a conspecific^37^. In these experiments, the naïve observer rat experienced an initial increase in dopamine levels, followed by a decrease in the absence of an explicit reward. Similarly, Ripolles et al.^30, 38^ showed that when subjects successfully learn the meaning of new words presented in verbal contexts, in the absence of any explicit reward or feedback, they also experienced an increase in emotion-related physiological measures and subjective pleasantness ratings. Such learning in the absence of external reward or feedback was found to be causally related to synaptic dopamine availability^31^. When the subjects were provided with a dopamine precursor or a dopamine antagonist, both learning and pleasantness ratings were shifted as compared to a placebo group. The precursor group showed enhanced learning and pleasantness ratings, while the antagonist group showed a decrease in learning and pleasantness ratings.

Social interactions, themselves, can act as rewards^39–41^. Access to social stimuli or social isolation both recruit the dopamine reward system^42–44^. In the current study, we asked whether dopamine signaling contributes to social learning, defined here as the acquisition or facilitation of new skills by observation or exposure to a conspecific performing a well-defined behavior^45, 46^. We previously reported that naïve gerbils acquire a sound discrimination task significantly faster when exposed for 5 days to a demonstrator that was performing the task, as compared to three different control groups^46^. To test the hypothesis that social learning engages a dopamine-dependent reward signal that facilitates the subsequent acquisition of an auditory task, we used pharmacological loss- and gain-of-function manipulations of D1/D5 dopamine receptors in naïve observer gerbils during exposure sessions. Furthermore, to assess the behavioral signals that might contribute to social learning, we monitored the vocalizations and movements of both observer and demonstrator during the exposure sessions. Together, the results suggest that dopaminergic signaling is both necessary and sufficient to facilitate socially-mediated task acquisition and one important social cue is the demonstrators’ vocalizations at trial initiation.

## Results

### Presence of an explicit reward improves social learning

Our first objective was to determine whether an explicit reward could facilitate social learning. As described previously^46^, demonstrator gerbils were first trained by an experimenter to perform a Go-Nogo amplitude modulation (AM) rate discrimination task. Briefly, the demonstrators were placed on controlled food access and trained to initiate each trial by placing their nose in a nose port. The Go stimulus (12-Hz AM noise) indicated the presence of a food reward at the food tray, while the Nogo stimulus (4-Hz AM noise) signaled the absence of a food reward. A discrimination performance metric, d-prime (d′) was calculated for each session as d′ = z(hit rate) − z(false alarm rate). Once animals performed the task with a d′ > 1.5, they qualified as a demonstrator (for more details, see “Methods” section).

**Figure 1:**
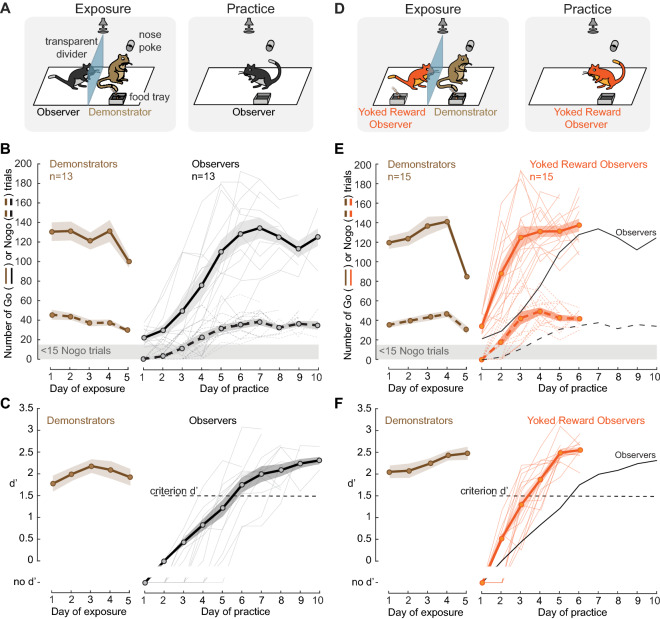
Yoking food reward to an observer facilitates social learning. (**A**) Replication of Fig. 1 from Paraouty et al.^46^. Experimental Design. Left: Naïve observer gerbil (black) was separated from a performing same-sex demonstrator gerbil (brown) by a transparent divider. The demonstrator was previously trained by the experimenters (see “Methods” section for details). Right: Following 5 days of exposure, the practice phase began during which the naïve observer gerbil was permitted to practice the task on its own. (**B**) Mean ± standard error of the mean (SEM) of the number of Go and Nogo trials performed by the demonstrators (5 males; age = 122.6 ± 4.7 days during the 5 days of exposure (brown), and by the naïve observer gerbils (age = 101.4 ± 4.1) during the practice sessions (black). (**C**) Mean ± SEM d′ values of the demonstrators during the 5 days of exposure (brown) and of the naïve observer gerbils during the practice sessions (black). The performance sensitivity, d′ was calculated once the observers performed > 15 Nogo trials. (**D**) Experimental Design. Left: Yoked reward observer gerbil (orange) was separated from a performing demonstrator gerbil (brown) by a transparent divider. A food tray was also present in the observer’s compartment, and the food reward of the demonstrator animal was yoked to that of the observer. Right: Practice session, similar to A (right). (**E**) similar to B, with demonstrators (4 males; age = 130 ± 4.2) and yoked reward observers (age = 96.8 ± 5.3). (**F**) Similar to C for the yoked reward observers. For comparison purposes, the observers’ results from 1B and 1C are replicated on Figures (**E**) and (**F**), respectively.

Our previous results demonstrated that observation alone led to social learning^46^ and we first replicated that finding. A naïve observer gerbil was placed adjacent to a previously-trained and performing demonstrator gerbil for 5 consecutive exposure sessions (Fig. 1A, left, also see Video S1). The demonstrator compartment was separated from the observer compartment by a transparent divider and possessed a nose port and a food tray, thereby allowing the demonstrator gerbil to initiate and perform trials. The naïve observer gerbil had access to all sensory cues emanating from the demonstrator and to the Go and Nogo sounds delivered 1 m above the test cage. During each exposure session, the naïve observer gerbil was exposed to a minimum of 80 Go trials and 20 Nogo trials performed by the demonstrator (Fig. 1B, brown lines). Following the fifth exposure session, the demonstrator was removed as well as the divider and the naïve observer gerbil was permitted to practice the task on its own (Fig. 1A, right). The sensitivity metric, d′, was computed for all sessions during which the naïve observer gerbil performed > 15 Nogo trials. On average, the naïve observer gerbils required 5.6 ± 0.35 days (mean ± standard error) to perform the task at a criterion d′ of 1.5 (Fig. 1C, black lines). No significant difference was found between the number of days taken to reach the criterion d′ by the current observers and those tested previously^46^, Fig. 1, Wilcoxon rank sum test, X^2^(1) = 0.27, *p* = 0.601). Therefore, the current study contains a replication of our previous finding that social learning facilitates acquisition of a sound discrimination task^46^, as compared to 3 control conditions that were examined in the original study: (1) naïve gerbils deprived of any prior exposure before performing the task, (2) naïve gerbils exposed to the test cage alone for 5 days prior to performing the task, and (3) naïve gerbils exposed to a non-performing demonstrator for 5 days prior to performing the task. The 3 control groups all took significantly longer to perform the task and reach the criterion d′ of 1.5 as compared to the observer groups (see Fig. 2 in Ref.^46^).

To test whether an explicit food reward could further enhance social learning, we next placed a food dispenser in the observation chamber (Fig. 1D, left) and provided naïve observers with a food reward that was yoked to the demonstrator’s performance during the five exposure sessions. In other words, a food pellet was delivered to both the demonstrator and the naïve observer when the demonstrator responded accurately on Go trials. The naïve observers in this case were also exposed to a minimum of 80 Go trials and 20 Nogo trials in each pairing session (Fig. 1E, brown lines). On average, the naïve observer gerbils with an explicit yoked reward required 3.3 ± 0.21 days to perform at criterion (Fig. 1F, orange lines) which was significantly faster, as compared to observation alone (Wilcoxon rank sum test, X^2^(1) = 12.87, *p* = 0.0003). Therefore, an explicit food reward during the exposure sessions facilitates social learning.

### Decreasing dopamine receptor signaling diminishes social learning

**Figure 2:**
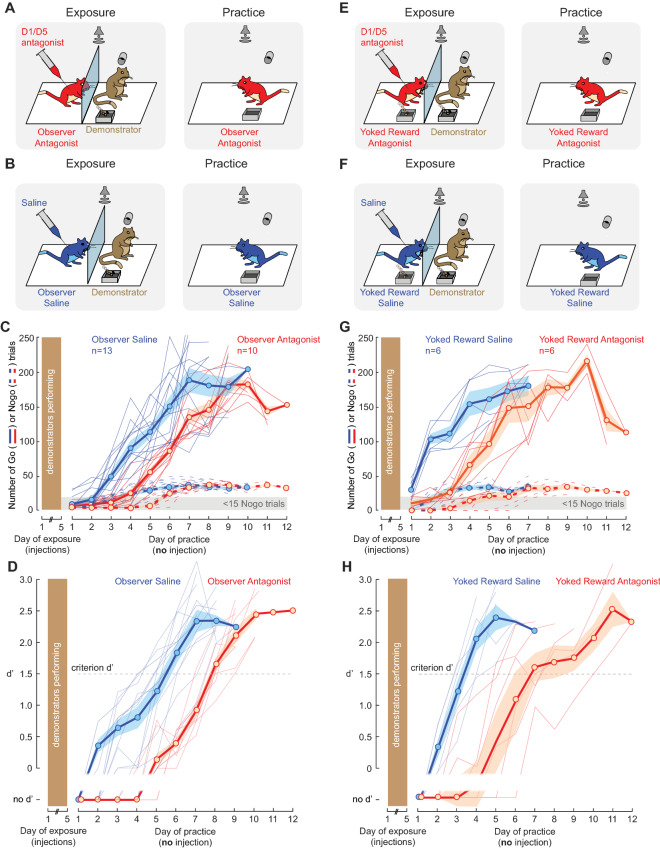
Decrease of tonic dopamine level degrades social learning. (**A**) Prior to each exposure session, the naïve observer gerbils were given an injection of a dopamine receptor antagonist (5 males; age = 99.5 ± 8.6 days; see “Methods” section for details). After 15–20 min of recovery, the exposure session began (left) with same-sex demonstrators (age = 138 ± 3.7). No drug was injected during the practice sessions (right). (**B**) Similar to **A**, except that saline was injected to the observers (5 males; age = 104.3 ± 10.7) prior to each exposure session (left) with the demonstrators (age = 143 ± 5.4). No drug was injected during the practice sessions (right). (**C**) Individual and mean ± SEM of the overall number of Go and Nogo trials performed by the observer gerbils during the practice sessions (antagonist-injected observers in red and saline-injected observers in blue). (**D**) Individual and mean ± SEM of the performance d′ of the observer gerbils as a function of practice sessions. (**E**) Prior to each exposure session, the yoked reward observer gerbils were given an injection of a dopamine receptor antagonist (4 males; age = 111.3 ± 8.9). After 15–20 min of recovery, the exposure session began (left) with the demonstrators (age = 166 ± 4.5). No drug was injected during the practice sessions (right). (**F**) Similar to **E**, except that saline was injected to the observers (5 males; age = 110.3 ± 8.5) prior to each exposure session (left) with the demonstrators (age = 119 ± 5.7). No drug was injected during the practice sessions (right). (**G**) Similar to **C** for the yoked reward observers. (**H**) Similar to **D** for the yoked reward observers.

Next, we asked whether an intrinsic dopaminergic signal during the exposure sessions contributed to social learning. Prior to each exposure session, the naïve observer gerbils were briefly anaesthetized with isoflurane and given an intra-peritoneal injection of a D1/D5 dopamine receptor antagonist, SCH-23390 (0.03 mg/kg, Fig. 2A, left) or an injection of saline (Fig. 2B, left). Animals were allowed to recover for 15–20 min before the exposure session began. Following the 5 days of exposure, the naïve observer animals were allowed to practice the task and no further injections were delivered (Fig. 2A-B, right). When D1/D5 receptors were blocked during the exposure sessions, naïve observer gerbils took significantly longer to reach the criterion performance, as compared to the saline-injected observer animals (Fig. 2D; Wilcoxon rank sum test, X^2^(1) = 13.81, *p* = 0.0002). The saline-injected animals did not differ significantly from the uninjected animals (from Fig. 1C; Holm–Bonferroni-corrected post-hoc comparisons, *p* = 0.7805), suggesting that neither saline nor the handling of the animal for the injection influenced the rate of task acquisition. During the first practice sessions, both trial initiation (Fig. 2C) and hit rates (Supplementary Figure S1D) were significantly poorer for the antagonist-injected animals as compared to all other groups (Holm–Bonferroni-corrected post-hoc comparisons, *p* < 0.05). This suggests that the dopamine receptor antagonist injections prior to the five exposure sessions led to a significant delay in task acquisition. In fact, the antagonist-injected animals did not initiate as many trials as the saline-injected observers during practice days 1–4 (Fig. 2C*; p* < 0.05). However, once the antagonist-injected animals were performing enough correct trials (hit rate > 25%), their mean hit rate improved considerably from 27.5% (practice day 4) to 86% (practice day 5). The d′ improvement from practice days 4 to 10 was similar to the overall d′ improvement of saline-injected observers (Fig. 2D*; p* < 0.05). Overall, a decrease in tonic dopamine level during the exposure sessions led to a significant decrease in the subsequent rate of task acquisition.

We also tested whether a decrease in intrinsic dopaminergic signal could be compensated for by the presence of an external food reward during the exposure sessions. Two additional groups of naïve observer gerbils were briefly anaesthetized prior to each exposure session and given an intra-peritoneal injection of the D1/D5 dopamine receptor antagonist, SCH-23390 (0.03 mg/kg, Fig. 2E, left) or an injection of saline (Fig. 2F, left). After recovery, both groups of naïve animals were provided with the exposure sessions, during which they received a food reward that was yoked to the demonstrator’s performance. Following the 5 days of exposure, the yoked reward observer animals were allowed to practice the task and no further injections were delivered (Fig. 2E-F, right). Naïve observer gerbils took significantly longer to reach the criterion performance when D1/D5 receptors were blocked, despite the presence of the yoked food reward during exposure sessions, as compared to the saline-injected control group (Fig. 2H; Wilcoxon rank sum test, X^2^(1) = 8.67, *p* = 0.0032). In addition, the saline-injected animals did not differ significantly from the uninjected animals with the yoked reward (from Fig. 1F; Holm–Bonferroni-corrected post-hoc comparisons, *p* = 0.8861), suggesting that neither saline nor the handling of the animal for the injection influenced the rate of task acquisition. Trial initiation (Fig. 2G) and hit rates (Supplementary Figure S1D) were significantly impacted during the first practice sessions for the antagonist-injected animals despite the presence of the yoked reward during the exposure sessions (Holm–Bonferroni-corrected post-hoc comparisons, *p* < 0.05). Thus, these results confirm that a decrease in intrinsic tonic dopamine level during the exposure sessions leads to a significant decrease in the subsequent rate of task acquisition despite the presence of yoked food reward. Furthermore, comparison of both observer groups that received the dopamine antagonist revealed that observers with the yoked food reward did not perform significantly better than the one without the yoked food reward (Holm–Bonferroni-corrected post-hoc comparisons, *p* = 0.0807). Together, these results suggest a crucial role of intrinsic dopamine signaling in social learning.

### Pharmacological increase in tonic dopamine level facilitates social learning

**Figure 3:**
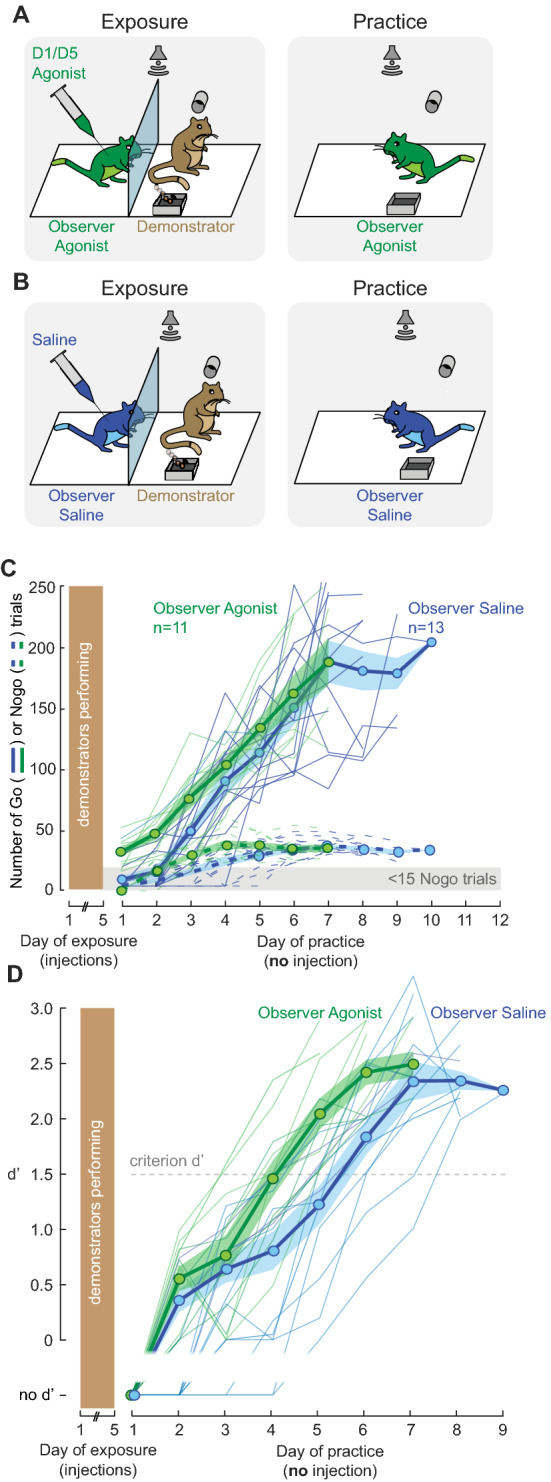
Increase of tonic dopamine level enhances social learning. (**A**) Prior to each exposure session, the naïve observer gerbils were given an injection of a dopamine receptor agonist (6 males; age = 100.3 ± 12 days; see “Methods” section for details). After 15–20 min of recovery, the exposure session began (left) with the demonstrators (age = 139 ± 5.2). No drug was injected during the practice sessions (right). (**B**) Similar to A, except that saline was injected to the observers (5 males; age = 104.3 ± 10.7) prior to each exposure session (left) with the demonstrators (age = 163 ± 5.4). No drug was injected during the practice sessions (right). (**C**) Individual and mean ± SEM of the overall number of Go and Nogo trials performed by the observer gerbils during the practice sessions (agonist-injected observers in green and saline-injected observers in blue). (**D**) Individual and mean ± SEM of the performance d′ of the observer gerbils as a function of practice sessions.

In order to test whether a high intrinsic dopamine level was enough to facilitate social learning, naïve observer animals received a D1/D5 dopamine receptor agonist during the exposure sessions. Prior to each exposure session, naïve observer gerbils were briefly anaesthetized and given either an intra-peritoneal injection of a D1/D5 dopamine receptor agonist, SFK-38393 (5.0 mg/kg, Fig. 3A, left) or an injection of saline (Fig. 3B, left). Animals were allowed to recover for 15–20 min before the exposure session began. Following the 5 days of exposure, the animals were allowed to practice the task and no further injections were delivered (Fig. 3A-B, right). When D1/D5 receptors were activated during exposure sessions, naïve observer gerbils subsequently learned the task significantly faster, as compared to the group of saline-injected animals (Fig. 3C; Wilcoxon rank sum test, X^2^(1) = 9.84, *p* = 0.0017). In addition, the agonist-injected observers reached the criterion d′ within a similar number of practice days as the yoked reward observers (from Fig. 1D; Wilcoxon rank sum test, X^2^(1) = 2.06, *p* = 0.1511). Together, these results suggest that an increase in intrinsic dopamine signaling during the exposure sessions led to enhanced task acquisition during the subsequent practice sessions.

### D1/D5 antagonist during exposure changes the behavioral pattern of the observer animals

Video footage from the five daily exposure sessions was recorded (see Video S1) and analyzed for a subset of observer animals (n = 5) in each of the seven experimental groups tested. To standardize the analyses, we only included the frames obtained during the first 20 min of each exposure session. Both the observer animal’s position (Supplementary Figure S2A) and its head orientation (Supplementary Figure S2C) were computed using DeepLabCut^47^ (see “Methods” section). On average, no significant difference was found between observer animals with or without a yoked reward (Supplementary Figure S2B, left, Holm–Bonferroni-corrected post-hoc comparisons, *p* = 0.7859) in terms of distance travelled during each exposure session. However, observer animals which received the dopamine antagonist moved significantly less as compared to observer animals which received either saline or a dopamine agonist (Supplementary Figure S2B, middle, *p* = 0.0192 and *p* = 0.0012). In contrast, observers that received the dopamine agonist did not differ significantly from the saline observer animals (*p* = 0.3424). Yoked reward observers with the dopamine antagonist also moved significantly less as compared to the yoked reward saline controls (Supplementary Figure S2B, right, *p* = 0.015).

The head orientation of all observer groups was preferentially towards the observer’s food tray, whether or not a food tray was present in the observer’s compartment during the exposure sessions. However, in the presence of the food tray, the yoked reward observers oriented their head significantly more towards the food tray as compared to observers without a food tray (Supplementary Figure S1D, left, *p* = 0.0011). Interestingly, observers with the dopamine antagonist which moved significantly less, also showed less preference to the food tray location as compared to observers with saline or with dopamine agonist (Supplementary Figure S1D, middle, *p* < 0.0001 for both). Similarly, the yoked reward observers which received the dopamine antagonist showed a significantly reduced preference to the food tray as compared to the saline controls (Supplementary Figure S1D, right, *p* = 0.0019). Although the dopamine antagonist impacted both locomotion and head orientation, the yoked reward observers with dopamine antagonist consumed a similar number of food pellets during the exposure sessions as the yoked reward observers with saline (*p* = 0.776) or the yoked reward uninjected observers (*p* = 0.053). In addition, for all observers in the yoked reward condition (uninjected, saline-injected, and antagonist-injected observers), no correlation was found between the mean number of pellets consumed by the observers during the five exposure days and the subsequent number of practice days taken to reach the criterion d′ (r = 0.17; *p* = 0.399). Similarly, no correlation was found between the mean proportion of head orientation towards the demonstrator (0°–180°, Supplementary Figure S2C, left) and the subsequent number of practice days taken to reach the criterion d′ for all experimental groups (r = − 0.06; *p* = 0.438). Furthermore, although the antagonist-injected observers moved significantly less as compared to their respective saline controls, no significant correlation was found between mean locomotion for all experimental groups across the five exposure days and the subsequent rate of task acquisition (r = − 0.61; *p* = 0.074). Together, these results confirm that neither the trend towards reduced food intake nor the reduced locomotion of the antagonist-injected observers influenced the subsequent rate of task acquisition during the practice sessions.

### The timing of vocalizations during exposure matches closely the onset of the sound stimuli

Audio recordings from the five daily exposure sessions were obtained and analyzed using DeepSqueak^48^ (see “Methods” section) for the same subset of animals used for video analyses (n = 5 in each of the seven observer groups tested). To standardize the analyses, we only included calls that were present during the first 20 min of each of the exposure session. Interestingly, both demonstrator and observer animals vocalized during the exposure sessions. In general, male observers displayed significantly longer call durations and lower principal frequencies, as compared to female observers (Supplementary Figure S3A; Steel–Dwass comparison, *p* < 0.0001 for both vocalization parameters). The dopamine receptor antagonist injections prior to the five exposure sessions significantly decreased the mean number of observer vocalizations, as compared to their respective saline controls (Supplementary Figure S3B; Wilcoxon comparisons for observers*, p* = 0.012; yoked reward observers, *p* = 0.037). The results also revealed a significant correlation between the mean number of observer vocalizations and demonstrator vocalizations (Pearson’s correlation coefficient, r = 0.37, *p* = 0.027). Thus, demonstrators paired with antagonist-injected observers vocalized significantly less as compared to demonstrators paired with agonist-injected observers and yoked reward observers (*p* = 0.022 for both comparisons). However, demonstrators paired with yoked reward observers which received the antagonist vocalized similarly as demonstrators paired with yoked reward and saline-injected observers (*p* = 0.296 and *p* = 0.210, respectively). We found a significant correlation between the mean number of demonstrator vocalizations across the 5 exposure sessions and the subsequent rate of an observer’s task acquisition: faster task acquisition occurred when the demonstrator vocalized more (r = − 0.71, *p* = 0.0001). A similar but non-significant trend was found between the mean number of observer vocalizations across the 5 exposure sessions and the subsequent rate of observer’s task acquisition (r = − 0.31, *p* = 0.067).

**Figure 4:**
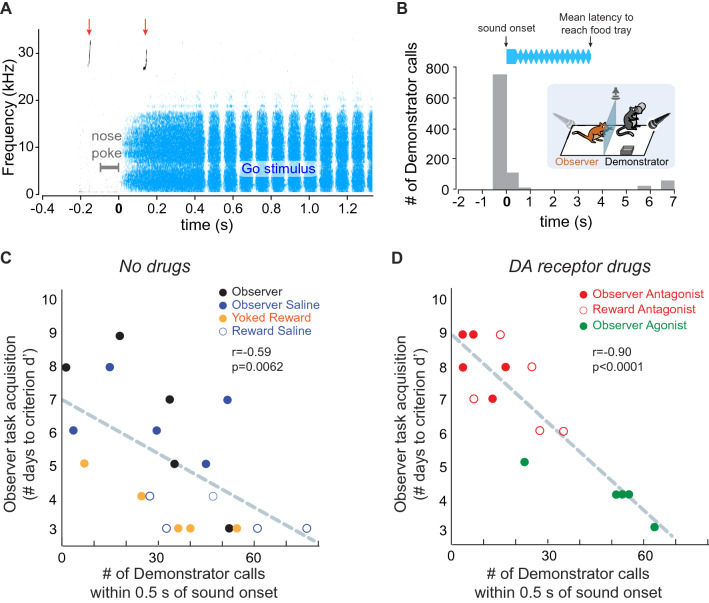
Time-locked demonstrator vocalizations with sound onset may provide key social cues to observers. (**A**) Example vocalizations of a demonstrator during an exposure session. (**B**) Histogram of time-locked demonstrator vocalizations as a function of the sound stimulus onset. (**C**) Mean number of time-locked demonstrator calls (within ± 0.5 s of the sound onset) plotted against the number of days taken by the respective observers to reach the criterion d′ of 1.5. Data are from the no-drug-treated demonstrator-observer pairs. (**D**) Mean number of time-locked demonstrator calls against the number of days taken by the drug-treated observers to reach the criterion d′ of 1.5.

For all exposure sessions analyzed, 60% of the demonstrators’ vocalizations were initiated around the time of trial initiation (i.e., nose-poke) and presentation of the Go or Nogo sound stimulus (Fig. 4B). While a similar temporal relationship was displayed by the naïve observers (Supplementary Figure S3D), these animals produced fewer vocalizations around the onset of the Go or Nogo sound stimulus (22% of all calls).

In contrast, very few demonstrator calls occurred around the time of food reward delivery (mean latency of response = 3.62 ± 0.39 s). Furthermore, while a majority of those time-locked demonstrators’ calls occurred prior to the nose poke and sound onset, the majority of the time-locked observers’ calls occurred after the sound onset. In addition, a majority of the time-locked demonstrators’ calls (~ 80%) were associated with a previous correct response on a Go trial (i.e., leading to a food reward).

To assess whether the abundance of the demonstrators’ vocalizations influenced the observers’ rate of learning, we asked whether there was a relationship between the proportion of time-locked demonstrators’ calls (defined as calls that occurred within ± 0.5 s of the sound onset) during the exposure sessions, and the observers’ subsequent rate of task acquisition during the practice sessions. When observer-demonstrator pairs from all groups were considered together, there was a strong negative correlation: a greater number of time-locked demonstrator vocalizations was associated with a smaller number of days for the observer to reach the criterion d′ (Pearson’s correlation coefficient, r = − 0.74, *p* < 0.001). To assess whether this effect was driven by our pharmacological manipulations, we separately analyzed groups that received no drug treatment, and groups that received the dopamine receptor agonist or antagonist. In fact, there was a negative correlation for observers that received no drug prior to the exposure sessions (Fig. 4C; Pearson’s correlation coefficient, r = − 0.59, *p* = 0.006). Similarly, a strong negative correlation was found for observers that received the dopamine receptor agonist or antagonist (Fig. 4D; r = − 0.90, *p* < 0.0001). In contrast, no correlation was found between the number of non-time-locked demonstrator calls (i.e., not within ± 0.5 s of the sound stimulus onset) and the subsequent learning rate of all observers (r = − 0.07, *p* = 0.690). Overall, these results suggest that the timing of the demonstrators’ vocalizations with the sound onset during the exposure sessions is a possible social cue for the observers to learn in this particular context.

### Non-task factors do not account for differences in learning rates

A range of experimental parameters could have contributed to the differences in learning rates measured across the different groups tested, including sex and postnatal age. For all groups tested, both male and female observers were used. No significant difference was found between male and female gerbils in terms of number of days to reach a criterion d′ of 1.5, when combined across groups (Kruskal–Wallis H test, X^2^(6) = 3.37, *p* = 0.770) or within each group (*p* > 0.05). The mean postnatal age of the different observer groups did not differ significantly (Kruskal–Wallis H test, X^2^(6) = 6.51, *p* = 0.369). No significant group difference was found in terms of total number of trials performed and d′ measures of the different demonstrator groups during the 5 exposure sessions (Kruskal–Wallis H test, X^2^(6) = 4.84, *p* = 0.564; X^2^(6) = 6.1, *p* = 0.423, respectively). Together, these results suggest that the differences in social learning rates could not be explained by non-task factors.

## Discussion

Dopamine signaling plays a broad role in learning that results in acquisition of explicit rewards^49^, suggesting that it may play a general role in social forms of learning that do not yield an immediate reward. To test this idea, dopamine signaling was manipulated only during the periods of social exposure. We first established that an explicit food reward during social exposure could facilitate the subsequent rate of learning (Fig. 1). We then tested whether a reduction in tonic dopamine signaling caused a reduction in social learning. We found that the rate of learning was significantly delayed when animals were treated with a D1/D5 receptor antagonist during social exposure (Fig. 2). Finally, to test whether tonic dopamine signaling was sufficient to facilitate social learning, in the absence of any external reward, animals were treated with a D1/D5 receptor agonist during social exposure (Fig. 3). This led to faster task learning, suggesting a role for dopaminergic signaling during social learning itself, but distinct from any dopaminergic signaling that may have occurred while animals practiced the task and received an explicit food reward. Overall, our findings suggest that theories of dopamine-dependent learning can account for task acquisition paradigms in which explicit rewards, and direct reward prediction errors, are unavailable.

Our results are broadly consistent with the role of dopaminergic signaling during social interactions. Indeed, dopamine has been found to play a vital role in social transmission of food preferences^50, 51^. For example, social learning of food preference is impaired when observer mice received a D1-type receptor antagonist^52^. Similarly, dopaminergic signaling is involved in learned aggression in rats^53^. Although the present study was not designed to assess the role of D1/D5 signaling on attention, it is plausible that the agonist led to enhanced arousal and attention during the exposure sessions^54–56^. In contrast, the antagonist may have decreased the general arousal and attention of the observers to both the social and non-social cues (to the demonstrator animal and the sound stimuli, respectively), or led to an aversive response to the test cage^57^ in addition to the decrease in internal dopamine signaling.

In our experiments, tonic dopamine levels in the naïve observers were modulated during each exposure session through systemic drug injections. However, dopamine signaling is often temporally precise at the moment when animals receive a reward or perceive a cue that predicts a reward^4^. Therefore, it is possible that our manipulations also enhanced or depressed the transient dopamine signals occurring during the exposure sessions. For example, increased firing of dopamine neurons in the observers could have been elicited by specific actions of the demonstrator, such as nose poking, or food acquisition, or vocalization. The yoked-reward observers in our study generally did not seek, nor consume, the food pellet immediately after its delivery during the exposure sessions. Therefore, it is plausible that both a general increase in dopaminergic activity, as well as a temporally precise signal (time-locked demonstrator vocalizations) could have elicited dopaminergic activity transients, and each served to facilitate an expectation for sound cues and potential rewards. Future work would require that dopamine neuron activity is monitored during the exposure periods to directly determine whether signaling is tonic or phasic.

Auditory forms of social learning have been particularly well-studied in songbirds^58–62^, where dopaminergic signaling is involved in both song learning and song production^63^. Indeed, Tanaka et al.^64^ showed that exposure to a live, singing tutor selectively activated dopamine neurons in a pupil bird. In contrast, the presence of a non-singing animal or a song playback failed to activate dopamine neurons. Importantly, song learning was completely blocked when dopamine fibers were eliminated prior to the exposure periods with the tutor. Similar mechanisms may operate in rodent auditory cortex. Local stimulation of D1/D5 receptors in rat and gerbil auditory cortex can boost sound detection, and dopamine afferent activity can induce long-term plasticity of tone-evoked responses^65–67^.

As shown in Paraouty et al.^46^, gerbils are able to use either visual or auditory cues during the exposure periods. Furthermore, they have access to olfactory cues which may heighten arousal and somatosensory cues which may permit adaptation to the general testing environment. Therefore, we suggest that observer animals are using these modalities, either individually or in combination during the course of a single exposure session, and learning could accrue from each. One intriguing possibility is that observers attend to the demonstrators’ vocalizations which were closely linked to a behavior (nose poke) and the sound stimulus onset (Fig. 4). It is possible that those time-locked demonstrator calls could reflect a recently received food reward (80% of calls were associated with a reward in the previous trial), or anticipation of a future reward that is associated with trial initiation. We further speculate that the timing of the demonstrators’ calls could boost the observers’ attention to both the nose-poke behavior and the sound stimuli, thereby promoting the formation of visual memories of the demonstrator’s position near the poke, or auditory memories of a sound cue associated with a vocalization, or both.

In the present study, the task to be learned through social exposure involved several discrete behaviors: trial initiation by nose poking, reward-seeking following a Go stimulus, and the withholding of reward-seeking following a Nogo stimulus. The movements of the naïve observer gerbils during the exposure sessions indicate some nascent forms of imitation, with a preferred head direction towards the food tray location (Supplementary Figure S2C,D), suggesting one element of the task that may be learned early on, even in the absence of visual cues^68^. This head orientation preference may be explained, at least in part, with (1) the cage design that was less wide than long, (2) the lateral position of rodent eyes which permit attention while at right angle to visual objects, and (3) the gerbil’s ability to attend to auditory cues^46^. However, although administration of D1/D5 antagonist significantly impacted locomotion (see Supplementary Figure S2B), the general amount of movement or head direction during the exposure sessions were not correlated with the subsequent performance of the observers.

The current results do not exclude the participation of other neuromodulatory circuits. When learning from conspecifics, the observer’s attentional and memory resources are focused on the demonstrator’s exploration and performance of skilled behaviors^69^. Therefore, it is likely that the cholinergic and noradrenergic systems play prominent roles in the modulation of attention, arousal, memory formation^70, 71^. Moreover, social interactions during the exposure sessions likely engage oxytocin and serotonin signaling^40^. Indeed, oxytocin has been shown to play a key role in auditory learning in a social context^72^. Ultimately, it will be necessary to assess the relative impact of each mechanism on the neural plasticity that supports social learning.

## Methods

### Experimental animals

Gerbil (Meriones unguiculatus, n = 130) pups were weaned at postnatal day (P) 30 from commercial breeding pairs (Charles River). Littermates were caged together, but separated by sex, and maintained in a 12 h light/dark cycle. All procedures related to the maintenance and use of animals were approved by the University Animal Welfare Committee at New York University, and all experiments were performed in accordance with the relevant guidelines and regulations.

### Behavioral setup

The behavioral setup was similar to Paraouty et al.^46^. Gerbils were placed in a plastic test cage (dimensions: 0.4 × 0.4 × 0.4 m) that was housed in a sound attenuation booth (Industrial Acoustics; internal dimensions: 2.2 × 2 × 2 m), and observed via a closed-circuit monitor. Auditory stimuli were delivered from a calibrated free-field tweeter (DX25TG0504; Vifa) positioned 1 m above the test cage. Sound calibration measurements were made with a ¼ inch free-field condenser recording microphone (Bruel & Kjaer). A pellet dispenser (Med Associates Inc, 20 mg) was connected to a food tray placed within the test cage, and a nose port was placed on the opposite side. The nose port and food tray were equipped with IR emitters and sensors (Digi-Key Electronics; Emitter: 940 nm, 1.2 V, 50 mA; Sensor: Photodiode 935 nm 5 nS). Stimuli, food reward delivery, and behavioral data acquisition were controlled by a personal computer through custom MATLAB scripts and an RZ6 multifunction processor (Tucker-Davis Technologies).

### Stimuli

For the sound discrimination task, the Go stimulus consisted of amplitude modulated (AM) frozen broadband noise tokens (25 dB roll-off at 3.5 kHz and 20 kHz) with a modulation rate of 12 Hz and a modulation depth of 100%. The Nogo stimulus was similar to the Go stimulus, except for the modulation rate which was 4 Hz. Both Go and Nogo stimuli had a 200 ms onset ramp, followed by an unmodulated period of 200 ms which then transitioned to an AM stimuli. The sound level used was 55 dB SPL.

### Video recordings

Videos of the test cage were captured with a Logitech c270-HD webcam (30 frames per second, Best Buy). We used the open source software: DeepLabCut^47^ on a Windows 10 machine (Dell Precision 5820, 64-bit operating system) to track the gerbil’s position in the test cage during practice sessions. The network was trained with 1,030,000 iterations using a total of 1064 labeled frames (labeling of nose, left ear, right ear, and tail base) and tested on a set of 200 frames. Manual evaluation of labeling accuracy was achieved by comparing the labels acquired from the network on the test set with the manual labels.

### Sound recordings

Audio recordings were captured with two Dodotronic microphones (Ultramic 384K_BLE), placed on either side of the test cage. We used the open source software: DeepSqueak^48^ on a Windows 10 machine (Dell Precision 5820, 64-bit operating system) to track the vocalizations of both the demonstrator animal and the observer animal in each session. DeepSqueak-screener was used to first create a library of gerbil calls. A detection network was then produced using this library of gerbil vocalizations. All audio recordings were analyzed using this custom gerbil detection network, followed by a post-hoc denoising stage and manual confirmation of all individual calls. Due to the physical barrier (i.e., the divider) separating the observer and demonstrator animal, only a subset of calls were caught by both speakers. Thus, the majority of calls (85% on average across all sessions) of the demonstrator was only caught by the demonstrator’s microphone and the majority of calls (83% on average across all sessions) of the observer animal was only caught by the observer’s microphone. For the subset of calls that were caught by both microphones, the calls were attributed to the side with the highest power (in dB). For statistical purposes, we calculated the mean number of calls per animal across the 5 exposure sessions, although data from individual sessions are plotted in Supplementary Figures S3A–C.

### Experimenter trained demonstrator gerbils

Demonstrator gerbils (n = 43) were trained by the experimenters on a sound discrimination task. The demonstrators were placed on controlled food access prior to the start of training, and all animals were trained using an appetitive reinforcement operant conditioning procedure^46^. Animals first learned to approach the food tray and receive food pellets (Bio Serv) when the Go stimulus (12 Hz AM noise) was played. Animals were then trained to reliably initiate Go trials independently by placing their nose in the port (nose-poke duration: 0.1–0.15 s). Once animals were performing a minimum of 80 Go trials with a hit rate > 80%, Nogo trials were introduced. The probability of Nogo trials was kept at 30% in order to keep the animal motivated to perform the task. Nogo trials were paired with a 4-s time-out during which the house lights were extinguished and the animal could not initiate a new trial. The presentation of Go and Nogo trials were randomized to avoid animals developing a predictive strategy. For more details on the experimenter training procedure, see^46^.

Responses were scored as a hit when animals approached the food tray to obtain a food reward upon Go trials. If animals re-poked or did not respond during the 5-s time window following a Go stimulus, it was scored a miss. During Nogo trials, responses were scored as a false alarm when animals incorrectly approached the food tray. If animals re-poked or did not respond during the 5-s time window following a Nogo stimulus, then it was scored a correct reject. Hit and false alarm rates were constrained to floor (0.05) and ceiling (0.95) values to avoid d′ values that approach infinity. A performance metric, d prime (d′) was then calculated for each session by performing a z-transform of both hit rate and false alarm values: d′ = z(hit rate) − z(false alarm rate)^73^. To qualify as a demonstrator, animals were required to perform the task with a d′ > 1.5.

### Social learning paradigms

During each *exposure session* (see Video S1), a demonstrator gerbil performed the discrimination task in the presence of a naïve, untrained observer gerbil. No animals were excluded from the study. As shown in Paraouty et al. (Supplementary Figure S4)^46^, we found no significant difference between observers that were paired with either same-sex cagemates or same-sex non-cagemates. Thus, in the current study, we used both cagemates and non-cagemates demonstrators. In addition, the age difference between observer and demonstrator was identical for cagemates, or slightly older demonstrators for non-cagemates. As in Paraouty et al.^46^, we did not observe a significant correlation between the demonstrator’s performance nor age and the observer’s subsequent performance on the task.

Both the demonstrator and the naïve observer gerbil were placed on controlled food access. A divider (acrylic sheet) was placed within the test cage to separate the demonstrator compartment from the observation or exposure compartment^46^. The naïve observer animal could thus see the demonstrator perform the task and was also exposed to the Go and Nogo sound stimuli as the speaker was located 1 m above the test cage. For the observers, a nose port and food tray were present only on the demonstrator’s compartment, allowing the demonstrator to initiate and perform trials (Fig. 1A, left). For the yoked reward observers, a food tray was also present in the observer’s compartment, and the food reward of the demonstrator animal was yoked to that of the observer (Fig. 1D, left). In both conditions, the naïve observers were exposed to a minimum of 80 Go trials and 20 Nogo trials performed by the demonstrator gerbils in each exposure session.

After five daily exposure sessions, the divider was removed after the final day of exposure and the naïve observer gerbil was then allowed to practice the task (*practice session*). The observer’s food tray (when present, i.e., for the yoked reward conditions) was also removed during the practice sessions. During the first and second practice sessions, the observer gerbil was given the benefit of no more than five experimenter-triggered Go trials. These experimenter-triggered Go trials were initiated only when an animal was touching the nose port. This method of manually initiating Go trials was identical to the one used to train demonstrators, in order to maximize the animal’s interest in the nose port object. Except for these experimenter-triggered Go trials, all Go trials were initiated by the gerbil. In order to limit a source of high variance, we only introduced Nogo trials once an observer animal was reliably initiating Go trials and performed > 25 Hits. False alarm trials were paired with a 2-s time-out on the second day of Nogo trial introduction. For all following practice days, a 4-s time-out was used when animals false alarmed. A d′ was computed for all practice sessions during which a minimum number of 15 Nogo trials were presented^46^. This was the standard procedure used for all observer groups tested here.

### Drug and saline injections

Prior to each exposure session, a subset of naïve observer gerbils was briefly anaesthetized with isoflurane and given an intra-peritoneal injection of either physiological saline (0.9% NaCl) or a D1/D5 dopamine receptor antagonist, SCH-23390 (0.03 mg/kg, volume = 0.06 ml) or a D1/D5 dopamine receptor agonist, SFK-38393 (5.0 mg/kg, volume = 0.1 ml). In addition, a subset of yoked reward observer gerbils was injected with physiological saline (0.9% NaCl) or the D1/D5 dopamine receptor antagonist, SCH-23390 (0.03 mg/kg, volume = 0.06 ml). The doses were initially based from previous studies in gerbils^74–76^. Following injections, animals were allowed to fully recover in a clean recovery cage (for 15–25 min) before the exposure session began. Higher doses of both the agonist and antagonist produced noted behavioral and motor effects (excessive grooming and hyperactivity for the agonist, and reduced motor behavior for the antagonist).

### Performance measures and statistical analyses

Due to limited litter sizes, a given litter could not be split into all conditions. However, animals from one given litter was used for at least 2 conditions. In addition, for all groups, data was collected from at least 3 different litters in order to avoid any litter-specific biases. No litter differences were observed during the experimenter-training stages of the demonstrator animals. A performance measure (d′) was calculated for each animal: d′ = z(Hit rate) − z(False Alarm rate). Hit and False Alarm rates were constrained to floor (0.05) and ceiling (0.95) values. To avoid high variance, d′ was only computed for sessions in which the observer gerbil performed > 15 Nogo trials. For the computation of the mean d′ line, we used all values of d′ and attributed a zero to all NaN values of d′ (i.e., when an animal was initiating < 15 Nogo trials, hence no d′ value could be computed). However, for all statistical tests and for the computation of the mean number of days taken by each experimental and control group to reach a criterion d′ of 1.5, only actual d′ values were used. We first checked whether the number of days for each group to reach a criterion performance d′ of 1.5 were normally distributed using the Shapiro Wilk test of normality. As the latter were not sufficiently Gaussian, we chose to perform non-parametric tests. All group level statistical tests and effect size calculations were performed using JMP Pro 14.0 on a Mac platform. Comparisons were carried out using the Wilcoxon rank sum test, as indicated. For comparisons of all groups tested, one-way ANOVAs were computed followed by post-hoc multiple comparisons analyses, and alpha values were Holm–Bonferroni-corrected.

### Ethical approval

The authors confirm that the study was carried out in compliance with the ARRIVE guidelines.

## Supplementary Information

Supplementary Figures.

Supplementary Video S1. Social learning of an auditory discrimination task. A naïve *observer* gerbil was permitted to experience a trained conspecific *demonstrator* perform an auditory Go-Nogo discrimination task. The naïve observer gerbil was exposed to a minimum of 80 Go trials and 20 Nogo trials in each exposure session. The performance of the demonstrator during those 5 exposure sessions is shown in Figure 1 (brown lines). Following five such exposure sessions, the naive observer gerbil was subsequently permitted to practice the task. The performance of the observer gerbil during the subsequent practice sessions is shown in Figure 1 (black lines). The markings on top of the demonstrator and the observer gerbil were obtained using DeepLabCut with 4 labelled features marked: nose tip, left ear, right ear, and tail base.
