# Supplementary material for: Dopaminergic signaling supports auditory social learning
Source: Sci Rep. 2021 Jun 23;11:13117. doi: 10.1038/s41598-021-92524-1 (PMC8222360; doi:10.1038/s41598-021-92524-1)
Supplement: Supplementary file 1 — Supplementary Figures. [file 41598_2021_92524_MOESM1_ESM.pdf]

Title Dopaminergic signaling supports auditory social learning

Authors Nihaad Paraouty, Catherine R. Rizzuto, Dan H. Sanes

Supplementary Information

Supplementary Figures 1-4 and Figure legends

Supplementary Figure 1

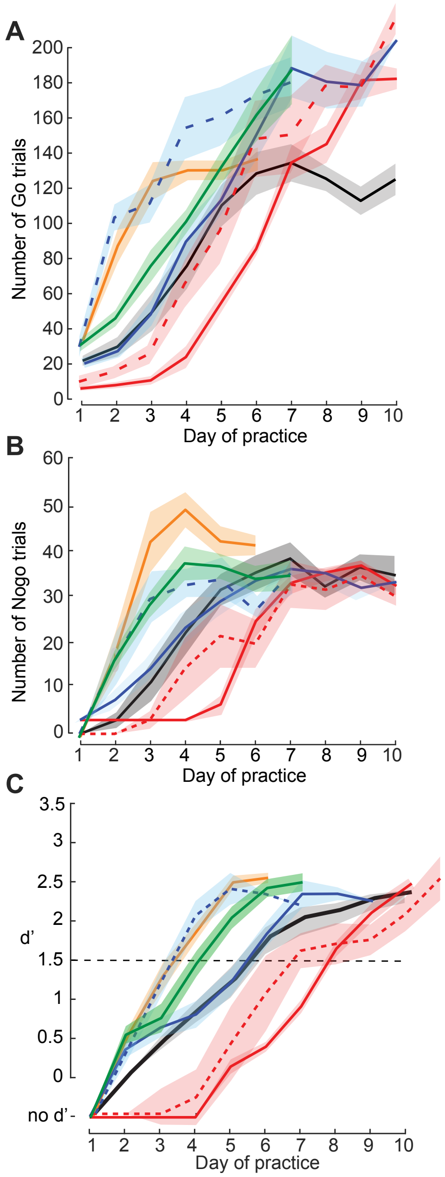

Comparison of behavioral performance of all experimental groups across practice days.
A. Mean number of Go trials ± SEM initiated by all observers in the seven different experimental groups of observers across practice days. **B.** Mean number of Nogo trials ± SEM initiated across practice days. **C.** Mean d' ± SEM across practice days. **D.** Mean hit rate ± SEM across practice days. **E.** Mean false alarm rate ± SEM across practice days. Mixed-model ANOVAs were used to reveal significant group differences, followed by Holm-Bonferroni-corrected post-hoc comparisons.

Supplementary Figure 2

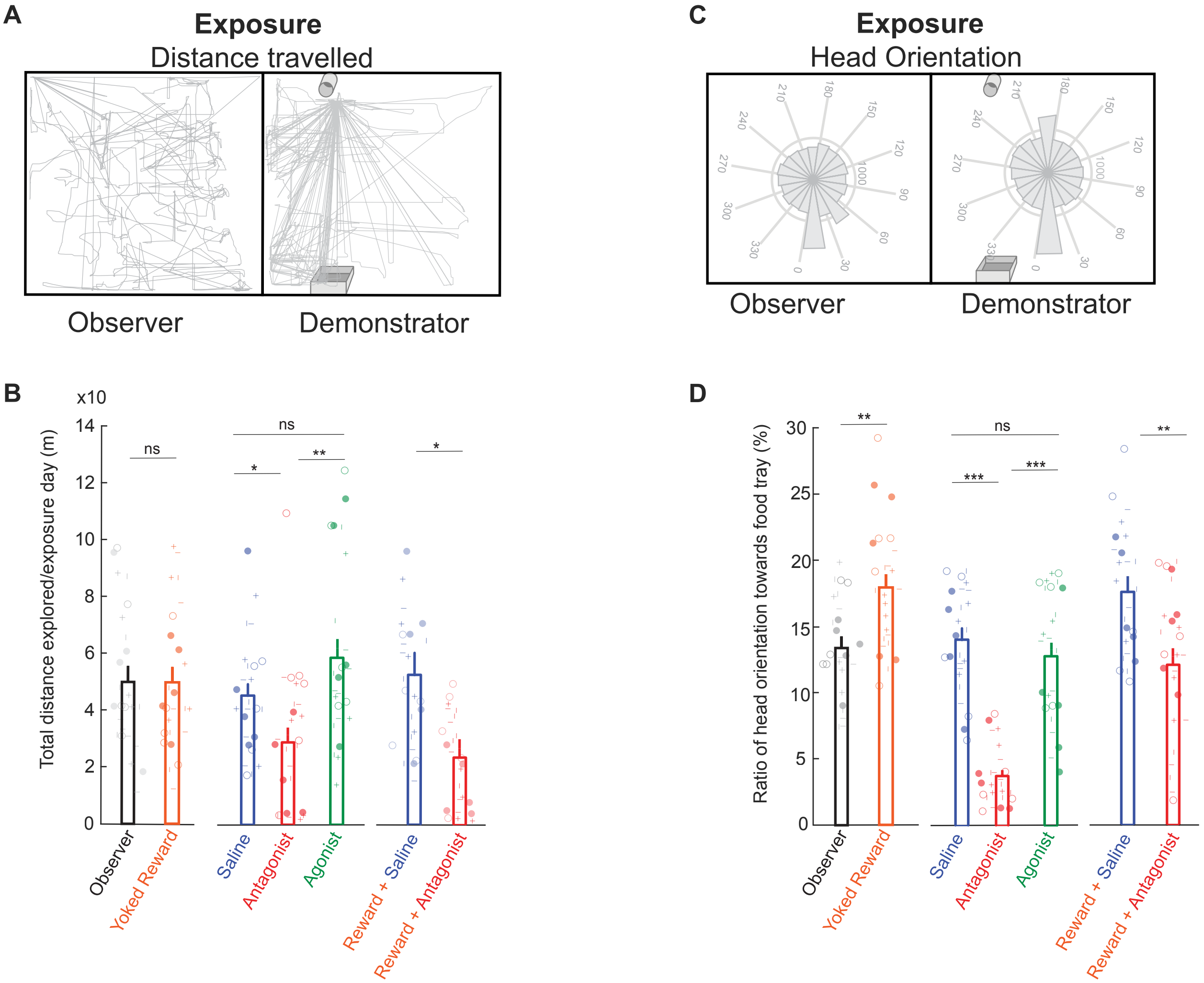

Administration of dopamine antagonist impacts the general behavior of observers.

A. Illustration of the distance travelled by a demonstrator and an observer during 1 example exposure session. **B.** Mean distance explored per exposure day for a subset of 5 observers in each of the different observer groups: left- naïve observers (black) and yoked reward observers (orange); middle- observers which received saline (blue), or a dopamine antagonist (red), or a dopamine agonist (green); right- yoked reward observers which received saline (blue), or a dopamine antagonist (red). The analysis of distance travelled was performed on the same number of frames (first 20 minutes) of each of the five daily exposure sessions. **C.** Illustration of the head orientation of a demonstrator and an observer during 1 example exposure session. **D.** Mean ratio of head orientation for the 0-18° which corresponds to the food tray for the same subset of 5 observers (as in B). Asterisks denote statistically significant post-hoc differences at the following levels: * p<0.05 and **p<0.01.

Supplementary Figure 3

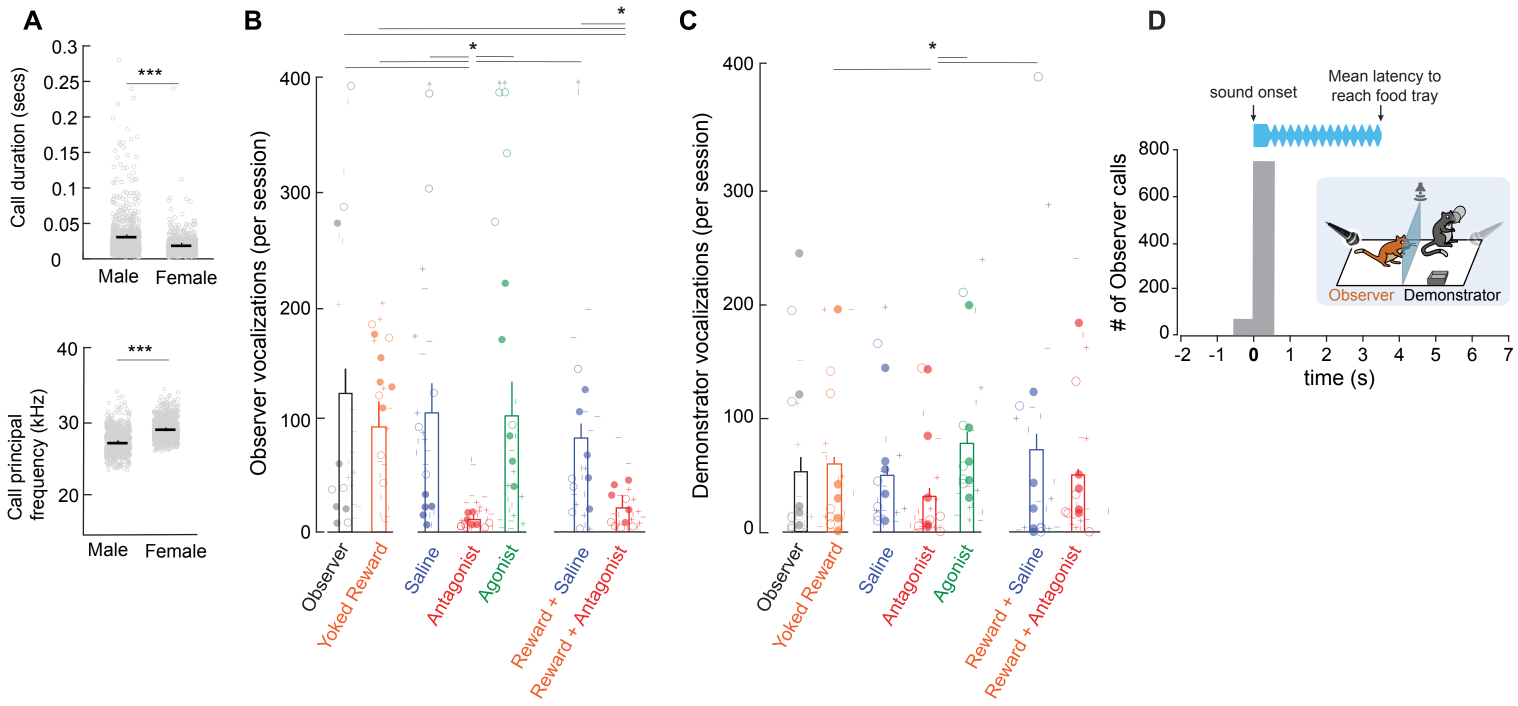

Administration of dopamine antagonist impacts observer vocalizations.

A. Sex differences in terms of call duration (top) and call frequency (bottom) for all observers and demonstrators. **B.** Mean number of calls of 5 observers (shown in different symbols) in each of the seven observer groups during the first 20 minutes of the audio recordings for each of the five daily exposure sessions. **C.** Mean number of calls of 5 demonstrators (same demonstrator-observer pairs in B and C) during the first 20 minutes of the audio recordings for each of the five daily exposure sessions. **D.** Histogram of time-locked observer vocalizations (22% of all observers vocalizations) as a function of the sound stimulus onset.

Supplementary Figure 4

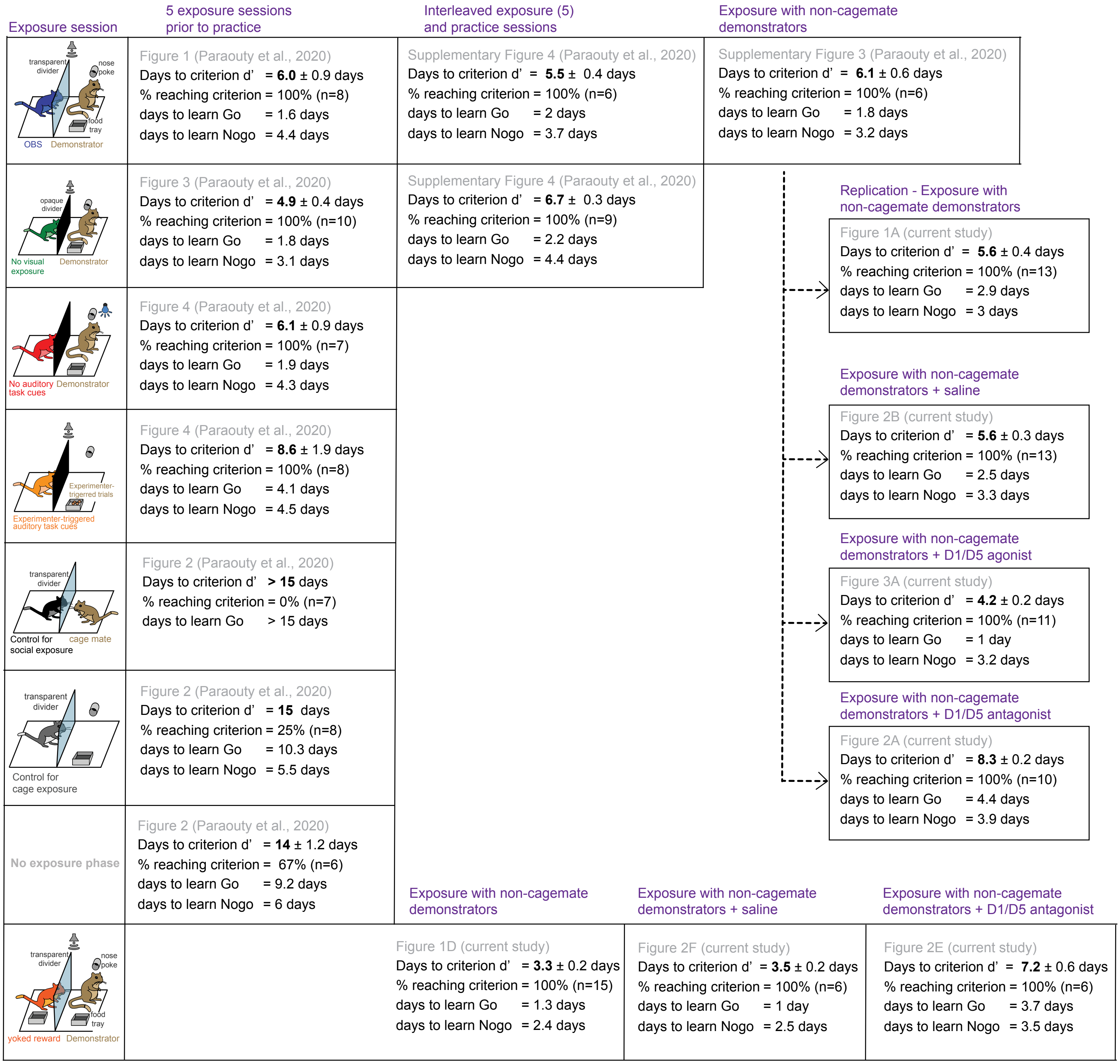

Summary of all social learning paradigms tested here and in Paraouty et al. (2020). Illustration of exposure sessions.
